# No evidence of decline in malaria burden from 2006 to 2013 in a rural Province of Gabon: implications for public health policy

**DOI:** 10.1186/s12889-015-1456-4

**Published:** 2015-02-04

**Authors:** Vanessa Assele, Gildas Ella Ndoh, Dieudonné Nkoghe, Thierry Fandeur

**Affiliations:** Unité de Parasitologie Médicale, Centre International de Recherches Médicales de Franceville, Franceville, BP 769 Gabon; Unité de Recherche et d’Analyses Médicales, Centre International de Recherches Médicales de Franceville, Franceville, Gabon

**Keywords:** Malaria trends, Rural Gabon, Morbidity, Slide positive rate, Ogooué-Ivindo province, Health Information System

## Abstract

**Background:**

The morbidity of malaria has steady declined in the urban regions of Gabon between 2000 and 2008, but caution should be exercised before generalizing this trend to the whole country because this finding has not been systematically confirmed in remote rural provinces.

**Methods:**

We conducted a retrospective survey using data on malaria cases recorded in North Eastern Gabon between 2006 and 2013 at health facilities in Makokou. Malaria data were analyzed, and associations with annual variations and patient age were assessed.

**Results:**

A global increase in clinical and confirmed malaria cases was observed over the study period. The rate of infection was significantly higher in children aged between 0 to 4 years than in children of 5 years and above, and in adults. Contrary to prior observations in urban and semi-urban areas of Gabon, malaria burden remained mostly unchanged or even increased in Makokou in the Ogooué-Ivindo province during these last 8 years.

**Conclusions:**

The persistence of *Plasmodium falciparum* pockets of sustained malaria transmission in rural Gabon may be related to an inadequate coverage of key interventions, to poor treatment seeking behavior and/or to a decline efficacy of treatments. Our results highlight the need to better adapt malaria control strategies to local epidemiological contexts and to environmental constraints. Equitable delivery of health service to hard-to-reach populations constitutes a challenging issue for the health authorities of Gabon.

**Electronic supplementary material:**

The online version of this article (doi:10.1186/s12889-015-1456-4) contains supplementary material, which is available to authorized users.

## Background

Malaria is a major cause of medical consultation and hospitalization in Gabon, and is responsible for about one-third of all childhood fevers [1]. Almost all episodes of malaria are due to *P. falciparum* and resistance to conventional antimalarial drugs is widespread [2,3]. Numerous studies have revealed the emergence of multidrug resistance among *P. falciparum*, including strains resistant to chloroquine, amodiaquine and sulfadoxine-pyrimethamine. As a result, the rate of therapeutic failure in Gabon since the early 2000s has been higher than 25%. The widespread resistance to sulfadoxine-pyrimethamine is worrying because this drug combination is still widely used in Gabon for the intermittent preventive treatment of pregnant women [4,5]. The susceptibility of parasites to antimalarial artemisinin derivatives and partners drugs such as mefloquine, amodiaquine, lumefantrine and piperaquine is poorly documented but low sensitivity to some drugs has nevertheless already been reported [5,6]. In 2009, 112,840 cases and 197 deaths attributable to malaria were recorded in Gabon, which roughly corresponds to an incidence rate of 77 per 1000 in the total population which certainly represents a low estimate of the malaria burden as a number of infections remain undiagnosed. The World Health Organization reported a rise in the incidence of malaria in Gabon over the past 15 years, with the exception of a decline between 2006 and 2008, which was followed by a surge in 2009 [7]. The statistical yearbook for Gabon reports a similar number of cases, although the figures do not demonstrate any decline over the same period with 123,708, 183,085, and 131,149 malaria episodes reported in 2006, 2007, and 2008, respectively [8].

The discordance in the reported statistics highlights the difficulty of obtaining an accurate evaluation and high-quality information in Gabon. In the absence of a nationwide system that routinely records malaria morbidity, trends are estimated by collecting data from a few selected public health facilities or sentinel sites located in, or adjacent to, urban areas such as the hospitals in Libreville, Port-Gentil, Lambaréné, and Franceville [9]. A substantial reduction in hospital admissions for malaria was reported in the predominantly urban coast of Gabon between 2000 and 2008, including a progressive change in malaria epidemiology. In particular, children >5 years of age became the most at-risk subpopulation. These improvements have been attributed to the implementation of expanded malaria control initiatives in Gabon from 2003, including intermittent preventive treatment of pregnant women, the distribution insecticide-treated bednets (ITNs), and increased availability of effective antimalarial drugs [10,11].

Presumably, these policy changes have significantly contributed to a reduction in malaria burden and hospital admissions; however, caution should be exercised before generalizing these trends to the whole country because these findings have not been systematically confirmed in remote rural provinces of Gabon. We sought to address this issue and investigate malaria burden and trends in malaria epidemiology in a rural region typical of Gabonese rainforest ecosystem; therefore, we collected and analyzed data on malaria cases reported in North Eastern Gabon between 2006 and 2013. Although a change in malaria epidemiology has been described over the last decade in urban areas of Gabon, our observations suggest that the malaria situation is mostly unchanged or even deteriorating in the interior of the country, despite the strengthening and diversification of preventive and curative interventions. We discuss the reasons for these differences in malaria risk according to economical and environmental constraints, and implications for better access to health services for hard-to-reach populations.

## Methods

### Study area

The study was carried out at Makokou, the administrative capital of the Ogooué-Ivindo Province, in North Eastern Gabon (0°33‘33“ N and 12°50’48" E). The Ivindo Valley is traditionally a major migration route for several ethnicities, including major groups in the North-East such as the Kotas, Fangs, and Kwélés. Baka Pygmies have been living in these forests for centuries. In addition to these four main ethnic groups, the majority of the merchants and traders in this region are immigrants from West Africa, Congo, and Cameroon. According to the “Direction Générale des Statistiques” du Gabon, the province displays one of the lowest population densities with less than 1.5 inhabitants per square kilometer. The Ogooué-Ivindo province had 48,862 inhabitants in the early 1990s (1993 census) and 64,163 inhabitants 10 years later (2003 census) indicating an annual growth rate of about 3.13% over the decade concerned. Based on this population growth rate and data reported in the 2003 census, this would give population estimates for 2006, 2007, 2008, 2009, 2011 and 2012 of 70,378, 72,581, 74,853, 77,195, 82,104 and 84,673, respectively, with an overall population of the province of about 77,000, for this period. The average incidence calculated over the study period and the annual incidences have been adjusted accordingly, on the basis of these demographic data. Ogooué-Ivindo is a landlocked region with poor economic development. Few industries are present except for wood logging, and the environment is exploited for both commercial and subsistence purposes. The average annual rainfall is approximately 1700 mm in Makokou. March–May and October–November are the rainiest months [12].

### Patients and data collection

Data on presumptive and confirmed malaria cases were retrospectively compiled from records archived at the outpatient department and laboratory at the Urban Health Centre and Regional Hospital of Makokou. Before the Regional Hospital became operational in 2011, the Urban Health Centre was the only health facility in Makokou. The Health Center, more central, nevertheless continued to receive patients after the hospital started activities, but attendance since steadily declined. For the purposes of the study, malaria cases reported at the Urban Health Center and Regional Hospital were merged, starting from 2011. Data were collected from outpatients who received consultations for febrile illness and were suspected of having malaria upon clinical examination (axillary temperature >37°5). When a patient was suspected of having malaria (then recorded as presumptive cases), a thick smear was prepared and stained with Giemsa, then examined for malaria parasites by conventional microscopy. The diagnosis was registered according to the patient’s ID and date of consultation. Malaria episodes (then recorded as confirmed cases) were defined according to the presence of fever >37°5 and clinical signs that were severe enough to warrant care, and a positive test results for asexual malaria parasites. Parasitemia threshold was not included in the definition of malaria episode because of the lack of accurate parasite counts. Instead, positive slides were semi quantitatively scored. As it is mostly the case in sub Saharan endemic countries, parasite densities were grossly determined and graded as “+” through “++++”. Because of the uncertainty in estimating parasite loads, this information was not included in our analysis. Similarly, infectious species were not formerly identified as microscopists routinely identify only *P. falciparum* in this area. Indeed, most malariologists agree that *P. falciparum* is responsible for >97% of infections in Gabon [13]. Data on patient sex and age were analyzed when available. All data were anonymously collected as part of routine analyses performed at Makokou health facilities, which are supervised by the Ministry of Health. This retrospective study is part of a larger survey on antimalarial drug resistance in Makokou that was initiated in 2013 (authorization N° 0-077/DGS from the Gabonese Ministry of Health). The hospital and health center record review was carried out in compliance with the Declaration of Helsinki 2000, following approval and ethical clearance from the National Ethical Review Committee of Gabon (N°0015/2013/SG/CNE).

### Data analysis

Presumptive and confirmed cases of malaria that were identified between January 2006 and March 2013 were processed for statistical analysis using Microsoft Excel. Data on presumptive and confirmed cases were separately reported on a weekly basis at health facilities, and were aggregated at monthly or annual intervals for analysis. Patient age has been systematically documented since 2009. Data collected in 2010 on clinical cases were incomplete and excluded from this analysis. Meteorological data were downloaded from Freemeteo (http://ga.freemeteo.com). Basic statistics and correlations were established using GraphPad software (http://www.graphpad.com/). Relative risks and Z scores were determined usingMedCalc (http://www.medcalc.org/calc/).

## Results

### Temporal variations in presumptive and confirmed cases of malaria

**Figure 1:**
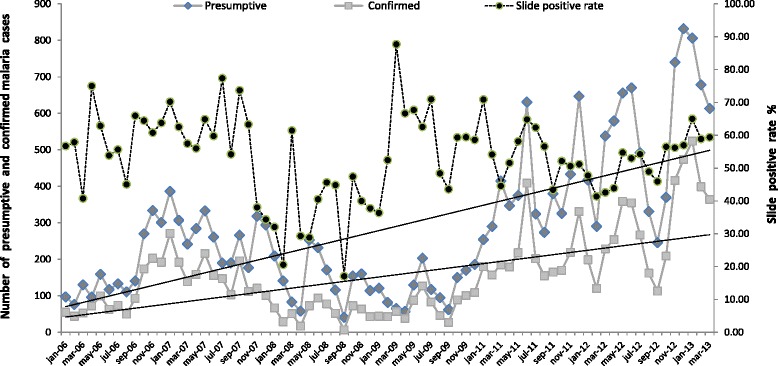
**Monthly variations in the number of presumptive and confirmed malaria cases and the slide positivity rate in Makokou between 2006 and 2013.**

Between January 2006 and March 2013, a total of 27,373 outpatients received consultations at Makokou Urban Health Centre and/or Regional Hospital, including 21,337 patients with malaria-like symptoms (presumptive cases). Of these, 11,600 patients tested positive for malaria (confirmed cases), but disease severity was not recorded. Figure 1 shows the variations in the monthly number of presumptive and confirmed malaria episodes that were documented over the study period. On average, 284.5 (95% confidence interval [CI] = 239.7–329.3) presumptive and 154.7 (95% CI = 181–128.4) confirmed cases were recorded per month, leading to an estimated annual incidence of 72.3 per 1000 (considering that the two main public health institutions in Makokou included in this study serve about one-third of the population of Ogooué-Ivindo Province and that the number of inhabitant in the Ogooué-Ivindo was of 77, 000, on average, for the study period). However, significant variations were noted from one year to another. The estimated incidence of confirmed malaria cases was of 50.0 per 1000 (n = 1173), 79.0 per 1000 (n = 1913), 25.9 per 1000 (n = 648), 33.1 per 1000 (n = 852), 93.9 per 1000 (n = 2570) and 111.0 per 1000 (n = 3157) in 2006, 2007, 2008, 2009, 2011, and 2012, respectively (*p* < 0,0001 Chi-square test for linear trend). We found a global and progressive increase in the number of presumptive (R = 0.647; *p* < 0.001) and confirmed (R = 0.581; *p* <0.001) cases in Makokou between 2006 and 2013, with a slight decline during 2008 and 2009; however, the rate of positive smears did not show any particular progressive trend (R = 0.07; *p* = 0.3) over the same period. The mean proportion of positive smears 53.6% (95% CI = 50.7–56.5) is consistent with a stable and sustained transmission of malaria in this region.

### Age-dependent morbidity

In total, 8183 patients were diagnosed by microscopy and treated for malaria between January 2009 and December 2011. The number of malaria cases in the age groups 0–4, 5–14, 15–50, and >50 years was 4296 (52.5%), 1451 (17.7%), 1930 (23.6%), and 506 (6.2%), respectively, over the period (Table 1). These proportions were stable between the various years and for all age groups (*p* > 0.3 for all permutations, Chi2 square test). The relative risk of malaria by age group compared to the risk of malaria in adults >50 years was 7.2 for children under the age of 5 years (*p* <0.0001) but remained identical in older age groups of 5–14 (1.6, *p* = 0.394) and 15–50 (1.0, *p* =1). Children between 0 and 4 years were between seven and nine times more likely to become infected than individuals of 5 years and older, and therefore represent the priority target group for preventive interventions.

**Table 1:** **Repartition of confirmed episodes of malaria by age and age-related risk of malaria in Makokou area in 2009, 2011 and 2012**

**Years**	**Age group (years)**	Population size estimate by age group*	**No. of confirmed malaria cases**	**Percent by age group**	**Annual incidence estimate ( 0/00)**	RR (95% CI)**	***p*** value***
2009	0-4	3860	1158	48.7	300.00	7.5 (2.7-20.5)	< 0.0001
	5-14	6176	391	16.4	63.31	1.5 (0.4-5.2)	0.519
	15-50	12351	679	28.6	54.98	1.2 (0.3-4.5)	0.733
	> 50	3345	150	6.3	44.84	Ref	-
2010	0-4	3981	1387	52.5	348.44	7.0 (2.8-17.1)	< 0.0001
	5-14	6369	491	18.6	77.09	1.6 (0.5-4.7)	0.394
	15-50	12738	595	22.5	46.71	1.0 (0.3-3.3)	1
	> 50	3450	169	6.4	48.99	Ref	-
2011	0-4	4105	1751	55.4	426.53	8.6 (3.6-20.8)	< 0.0001
	5-14	6568	569	18	86.63	1.8 (0.6-5.2)	0.27
	15-50	13137	656	20.7	49.94	1.0 (0.3-3.3)	1
	> 50	3558	187	5.9	52.56	Ref	-
2009-2011	0-4	3982	4296	52.5	359.63	7.2 (2.9-17.6)	< 0.0001
	5-14	6371	1451	17.7	75.92	1.6 (0.5-4.7)	0.394
	15-50	12742	1930	23.6	50.49	1.0 (0.3-3.3)	1
	> 50	3451	506	6.2	48.88	Ref	-

*RR: Risk of malaria by age group relative to risk of malaria in adults (>50 years).

**p-values were calculated using the Z-score and RR. A *p*-value <0.05 was considered significant.

***Population denominators for the various age groups used to estimate the age-related risk of malaria in the Ogooué-Invendo were deduced from the age-structure of the Gabon population (http://populationpyramid.net/fr/gabon/). The 2010 pyramid population shows that young children between 0–4 years represented 14.6%, and that higher proportions 24%, 47.2 and 13.2%, respectively, were found in age groups 5–14, 15–50 and >50.

### Seasonal variations

**Figure 2:**
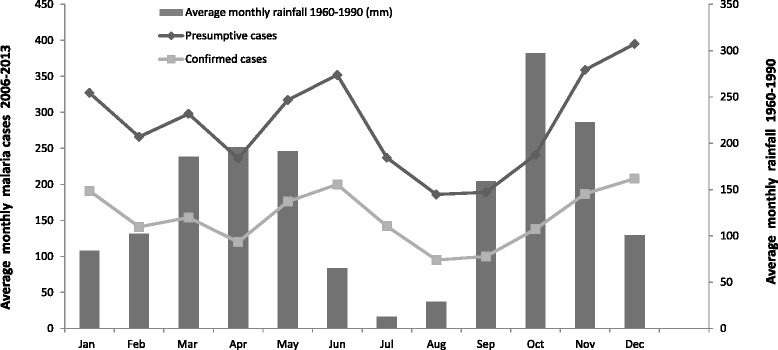
**Monthly distribution of the mean number of presumptive and confirmed episodes of malaria between 2006 and 2013 according to mean rainfall variations in Makokou from 1960 to 1990.**

The transmission of malaria is generally regarded as continuous in Gabon, although some important variations are observed according to region and season. Figure 2 shows the average monthly variation in the number of presumptive and confirmed cases between 2006 and 2013 in Makokou between 1990 and 2009 as a function of rainfall (rainfall data for 2010–2013 are unavailable). There are two rainy seasons: March–May and September–November. Rainfall is heaviest during the second period. Transmission peaks in June and December immediately after these rainy seasons, indicating that transmission is, at least to some extent, influenced by rainfall, even at this latitude.

## Discussion

This is the first large retrospective survey conducted on malaria morbidity in North Eastern Gabon. Regular collection of data on malaria prevalence and at-risk populations is mandatory to establish and implement health policies and to plan programs to fight malaria [14]. The identification of the populations and regions the most at risk depends on the quality and completeness of the recorded data. In Sub-Saharan Africa, this information is often lacking, either because the source documents simply do not exist or because they are confidential and cannot be easily accessed [15]. Data published over the last decade nevertheless indicate a 70% reduction in malaria morbidity in Ethiopia and Eritrea, and similar patterns have been reported in Kenya [15-17]. In West Africa, surveillance of healthcare facilities indicates a 50-85% decline in the rate of positive smears in Gambia, and encouraging trends indicating a decline of malaria have also been recorded in Mozambique, South Africa, Tanzania, Zambia, and Zanzibar [18-22]. In contrast, data on Central Africa are particularly scarce and show little change in the malaria situation relative to historical figures [23]. In Congo, the proportion of pediatric hospitalizations due to malaria (estimated at 30%) has remained stable [24]. Two cross-sectional studies performed in Cameroon in 2000 and 2004 indicate a slight reduction in the pediatric prevalence of asymptomatic carriage, but found no change in malaria prevalence in patients with fever [25]. In this respect, the malaria situation in Gabon appears more favorable. There is now increasing evidence that the epidemiological situation of malaria is changing in urbanized areas. The prevalence of malaria in children suffering from fever who were admitted to the Libreville hospital has dropped by almost 70% over the past 10 years, from 45% in 2000 to 15% in 2008. This reduction in malaria morbidity was accompanied by an increase in the age of the most at-risk group [10]. A similar trend was reported in hospitals in Franceville, located in southeast Gabon [26]. In this region, the number of children suffering from fever who tested positive for malaria significantly decreased from 68% in 2004 to 18% in 2010 [11]. The overall reduction in the incidence of malaria in Gabon and the decline in mortality have been attributed to the implementation of priority control interventions such as the use of artemisinin-derived antimalarial drugs and the distribution of ITNs [27,28].

Urban centers account for about 70% of the population of Gabon, but they do not reflect the health situation in more isolated regions where the infrastructure is poor, and where malaria is endemic and its prevalence is certainly under-estimated [29]. Despite the decline in malaria burden in many endemic areas, our data demonstrate that it has remained mostly unchanged and is even deteriorating in North-Eastern Gabon. The increase in malaria indices in Makokou therefore contrasts with the recent changes in malaria epidemiology reported in Libreville and Franceville, and is consistent with earlier observations in Central Africa pointing to a patchy pattern of malaria transmission according to geographic location. Congo, some rural settings in Angola, Central African Republic, Sudan, and some other neighboring regions, are areas where the prevalence of malaria is the highest. These areas inevitably serve as pockets of transmission, and a similar situation is likely to occur in Gabon [30,31]. As a consequence, these reservoirs of malaria infections should now become the main focus of malaria-targeted interventions [32].

The increase in the number of both presumptive and confirmed malaria cases, the stable rate of the percentage of positive smears (approximately 50%), and the higher risk of infection in young children between 1 and 4 years of age are consistent with a sustained transmission of malaria in the rural area of Makokou. Hence, the decline in malaria observed in urban and semi-urban coastal areas cannot be extended to the country as a whole. The medical technicians who screen for malaria and the methods used for diagnosis have remained mostly unchanged in health facilities in Makokou. Thus, it is unlikely that the difference in malaria prevalence is due to the poor performance of medical staff and microscopists who are mostly well trained and experienced. Changes in malaria incidence are also unlikely to be caused by the replacement of microscopy with a more sensitive approach such as rapid diagnostic tests (RDT). Indeed, the implementation of alternative methods for biological diagnosis is a top priority in Gabon but the use of RDT has been hindered by many challenging constraints for shipping and long-term storage of RDT in the field. As a result, the use of RDT remained rather limited in Gabon, at least until 2010. While there is no systematic quality control of slide readings in Gabon, we have confidence in the data for malaria cases reported because comparative surveys conducted since our study at the Makokou health facilities in 2012 and 2013 revealed a concordance rate of 76% (unpublished data) between microscopic examinations and RDT readings.

Malaria indices such as prevalence, incidence, and asymptomatic carriage are only slightly affected by seasonality in Gabon, and no major rainfall abnormalities were recorded in Gabon between 2000 and 2013 that could explain the malaria patterns observed [27,33]. Although annual variation in rainfall is modest in the Makokou region, the number of cases is twice as high in the peak seasons (May–June and November–December) than in off-peak seasons (February–April and July–September). This pattern is consistent with previous observations in regions where malaria is more seasonal: 1–2 months is required to re-establish larval habitats and for the re-initiation of the lifecycle of the parasite in host mosquitoes [34].

The epidemiological features and malaria trends observed in Makokou may be related to local factors. No demographic upsurge was apparent in the Ogooué-Ivindo Province during the study period. However, the construction of the hospital in 2011 possibly led to an increase in the frequency of medical consultations, and therefore in the number of malaria cases but, this element alone, cannot explain the differences in malaria trends we observed between Makokou and the Estuaire province as the number of cases increased from 1173 in 2006 to 3157 in 2012 in Makokou, corresponding to an increase by a factor of 5.3 over the period, whereas the population increased by a factor of only 1.2 over this period. Instead, the spread of resistance to conventional antimalarial drugs that continue to be used despite recommendations from the Ministry of Health, the uneven distribution of control and prevention methods across the country, and reluctance to seek treatment have undoubtedly contributed to the deterioration of the malaria situation over recent years. Artemisinin-based combination therapies (ACTs) for uncomplicated malaria were introduced in 2003 in Gabon, and the distribution of insecticide-treated mosquito nets started later on in 2005. The coverage of these interventions has however remained too limited to produce global and durable effects. In fact, ACTs were prescribed in only 24.5% of malaria cases in 2008 and in 52% of cases in 2010. Furthermore, the percentage of pregnant women receiving an intermittent preventive treatment (IPT) with sulfadoxine-pyrimethamine was largely unchanged between 2006 and 2010, remaining close to 60% for IPT1, and between 30% and 50% for IPT2. Between 2006 and 2010, only 323,586 ITNs were distributed for a national population of 1,556,222 in 2010. In 2010, the proportion of pregnant women who slept under an ITN was only 52% in the Ogouee-Ivindo Province, whereas in young children <5 years the proportion was 56% [9,28]. Incidentally, the decrease in malaria prevalence reported in Libreville and Port-Gentil between 2005 and 2008 was only observed later at Makokou, in 2008 and 2009 [7,10,11]. The delay in the decrease of malaria incidence in Makokou may presumably be a consequence of the late implementation of key health interventions. This finding is reminiscent of earlier studies showing that remote and underserved regions were the most affected by weakened efforts to combat malaria, due to inadequate funding, transport constraints and/or insufficient resources [35,36].

The analysis of malaria indicators is further clouded in Gabon by several confounding factors. Almost half of all cases are treated in private or informal sectors for which no statistics are available, suggesting that the incidence of *falciparum* malaria might be of about 200 per 1000 in the rural area of Makokou. Moreover, the current infrastructure is generally insufficient for the regular monitoring and follow-up of malaria patients. Many infections, although treated, are not confirmed by diagnostic tests. Finally, in the absence of a National Health Information System (NHIS) in Gabon, data are essentially collected from public hospitals in larger towns. The calculation of more accurate estimates of malaria burden in Gabon will require the implementation of screening programs, particularly in rural areas, to provide complete and representative values of various malaria indices, including incidence especially in children aged between 2 and 10 years [37]. The only national survey of this type carried out in Gabon between 2005 and 2011, reported a higher prevalence of infection in children <15 years old than in adults, and a higher risk of parasitic carriage in populations living in forest ecosystems [29]. Although these findings involve asymptomatic infections, they nonetheless illustrate the large heterogeneity of malaria endemicity in Gabon, and the divergent trends found in rural and urban areas.

## Conclusion

To conclude, contrary to prior observations in urban and semi-urban areas of Gabon, malaria burden has mostly remained unchanged, or even increased, in Makokou in the Ogooué-Ivindo province. The patchy distribution of malaria risk observed in the various provinces of Gabon highlights the limitations of global strategies, and the need to adapt malaria control programs and surveys to local epidemiological contexts. In particular, the implementation of a countrywide malaria diagnostic lab quality assurance program should be considered by health authorities to improve case investigation and reporting. Our results also underline the importance to establish a *bonafide* NHIS in Gabon. Such an initiative would strengthen health coverage and the monitoring of resistance to antimalarial drugs and insecticides. A NHIS would also improve the capacity of operational research and local programs to combat malaria by coordinating field interventions and the concerted actions of a number of actors: healthcare facilities, anti-malaria agencies, hospital clinicians, and researchers. The reduction of malaria burden observed in urbanized areas of Gabon should not obscure the sanitary situation that prevails in remote rural provinces. Equitable access to malaria prevention and treatment should therefore be priority for health authorities to consolidate gains, and extend the benefits of interventions to the country as a whole.

### Ethical approval

Ethical clearance was obtained from the National Ethical Review Committee of Gabon (N°0015/2013/SG/CNE) and was further authorized by the Gabonese Ministry of Health) authorization N° 0-077/DGS.
